# A Comprehensive Review Exploring Allergic Rhinitis With Nasal Polyps: Mechanisms, Management, and Emerging Therapies

**DOI:** 10.7759/cureus.59191

**Published:** 2024-04-28

**Authors:** Gowtham Narasimhan, Prasad T Deshmukh, Sagar S Gaurkar, Farhat Q Khan

**Affiliations:** 1 Otolaryngology, Jawaharlal Nehru Medical College, Datta Meghe Institute of Higher Education and Research, Wardha, IND

**Keywords:** precision medicine, immunomodulatory therapies, treatment, inflammation, nasal polyps, allergic rhinitis

## Abstract

Allergic rhinitis (AR) and nasal polyps (NP) are common inflammatory disorders of the upper airways that often coexist and significantly impact patients' quality of life. This comprehensive review explores the intricate relationship between AR and NP, elucidating the underlying mechanisms, clinical manifestations, and management strategies. Immunological mechanisms, genetic predispositions, and environmental factors contribute to the development and progression of both conditions. Pharmacological therapies, including intranasal corticosteroids and biologic agents, are cornerstone treatments for managing AR with NP. At the same time, surgical interventions such as functional endoscopic sinus surgery (FESS) may be necessary in refractory cases. Emerging therapies, including immunomodulatory agents and precision medicine approaches, hold promise in improving treatment outcomes. A multidisciplinary approach, personalized treatment plans, and patient education are essential for optimizing clinical practice. Future research should focus on identifying novel therapeutic targets, conducting large-scale clinical trials, exploring precision medicine approaches, and investigating the role of the microbiome. Addressing these research priorities and implementing evidence-based treatment strategies can improve outcomes for patients with AR and NP.

## Introduction and background

Allergic rhinitis (AR), commonly known as hay fever, is a prevalent allergic condition characterized by inflammation of the nasal mucosa in response to exposure to allergens such as pollen, dust mites, pet dander, or mold spores [1]. It affects millions worldwide and can significantly impair the quality of life due to symptoms such as nasal congestion, sneezing, itching, and rhinorrhea. AR can be seasonal or perennial, depending on the allergen triggers and individual susceptibility [1]. Nasal polyps (NP) are benign growths in the nasal passages or sinuses, often due to chronic inflammation. They are typically soft, noncancerous lesions characterized by swollen mucous membranes. They can obstruct the nasal airway, leading to symptoms such as nasal congestion, reduced sense of smell, facial pressure, and nasal drainage. Although the exact cause of NP is not fully understood, they are commonly associated with chronic rhinosinusitis, asthma, and aspirin sensitivity [2].

There exists a significant overlap between AR and NP, with studies suggesting that up to two-thirds of patients with NP also have concomitant AR [3]. The inflammatory processes involved in AR, including eosinophilic infiltration and release of inflammatory mediators, contribute to the development and progression of NP. Furthermore, AR may exacerbate symptoms and promote the recurrence of NP through ongoing inflammation and tissue remodeling [4]. This comprehensive review explores the intricate relationship between AR and NP, elucidating the underlying mechanisms linking these two conditions. This review seeks to provide valuable insights into managing AR with NP by examining the latest research findings, therapeutic approaches, and emerging treatments. Understanding the interplay between these disorders is crucial for optimizing patient care, improving treatment outcomes, and advancing our knowledge of allergic respiratory diseases.

## Review

Mechanisms of allergic rhinitis with nasal polyps

Immunological Mechanisms

IgE-mediated pathways: The IgE-mediated pathways implicated in AR entail a series of immune reactions initiated by the interaction between allergens and IgE antibodies bound to effector cells like mast cells and basophils. Upon allergen cross-linking with IgE antibodies on these cells, various mediators, including histamine, cytokines, and chemokines, are released, contributing to the hallmark inflammatory response seen in AR [5]. This cascade triggers an immediate mast cell response and activates T cells expressing Th2 cytokines such as interleukin (IL)-4 and IL-5, pivotal in IgE synthesis, eosinophil proliferation, and ongoing allergic inflammation [6]. Additionally, the binding of allergen-specific IgE to receptors on effector cells like FcεRI and FcεRII sets off an inflammatory cascade, resulting in the typical symptoms of AR [7]. Omalizumab, an anti-IgE antibody, has demonstrated promising outcomes in AR treatment by binding to free serum IgE, impeding its interaction with effector cell receptors and diminishing the associated inflammatory response [7]. Overall, the IgE-mediated pathways in AR occupy a central role in its pathophysiology, underscoring the significance of IgE in orchestrating allergic responses and the potential of targeted therapies like anti-IgE antibodies to manage AR effectively.

Inflammatory responses: The inflammatory responses in AR entail a sequence of immune reactions incited by allergens, culminating in symptoms such as nasal obstruction, rhinorrhea, nasal itching, and sneezing. This immune cascade is mediated by various cytokines, chemokines, and cells that sustain allergic inflammation, with notable involvement of eosinophilic infiltration and mast cells in conditions like NP and chronic rhinosinusitis [8,9]. The pathophysiology of AR manifests in a two-phase allergic reaction, commencing with an early-phase response featuring immediate symptoms like sneezing and rhinorrhea, followed by a late-phase response marked by nasal congestion and obstruction [9]. Effective therapeutic approaches for AR encompass medications such as second-generation antihistamines, intranasal corticosteroids, mast cell stabilizers, and leukotriene receptor antagonists like montelukast, which have exhibited promising outcomes in managing refractory cases [9]. Immunotherapy has also emerged as a viable treatment option for patients resistant to standard pharmacologic interventions, offering a means to modulate the immune system and potentially furnish long-term relief from allergic symptoms [9]. Overall, comprehending the inflammatory mechanisms underpinning AR is pivotal for formulating efficacious treatment strategies targeting symptomatic relief and the intricate immune responses implicated in the condition.

Genetic Predispositions

Genetic predispositions are known to significantly influence NP's pathogenesis, especially in AR. Research has provided evidence indicating a genetic basis underlying NP, with studies focusing on the expression patterns of specific genes associated with the nasal polyp phenotype [10]. Differential gene expression profiles have been identified between NP and normal nasal tissues, with efforts aimed at identifying susceptible genes linked to traits associated with NP [11]. Certain gene products, regulated at various levels such as transcription, mRNA processing, translation, and degradation, underscores the complex genetic mechanisms involved in the development and progression of NP in the context of AR [11]. Furthermore, investigations have shed light on the potential role of activated ras family genes and the influence of aspirin intolerance in patients with chronic rhinosinusitis and NP, providing insights into the genetic factors contributing to these conditions [11]. Overall, genetic predispositions play a crucial role in determining susceptibility to NP, impacting immune responses, inflammatory processes, and molecular alterations associated with the development of these conditions.

Environmental Triggers

Environmental triggers for AR encompass various substances capable of eliciting an exaggerated immune response, resulting in symptoms such as nasal congestion, itching, fatigue, headaches, watery eyes, and sneezing. Common environmental triggers include pollens from trees, grasses, weeds, mold spores, animal dander, and dust mites [12,13]. These triggers can be classified into two main categories: perennial allergens, which persist year-round and include indoor molds, animal dander, and dust mites, and seasonal allergens, which are more prevalent during specific times of the year and consist primarily of pollens and certain outdoor molds [13]. Irritants such as smoke, potpourri, perfumes, cleaning agents, solvents, incense, and soaps or detergents can also trigger AR [14]. Recognizing these environmental triggers is essential for effectively managing AR, and identifying specific allergens through testing can assist individuals in avoiding or minimizing exposure to these triggers.

Clinical presentation and diagnosis

Symptoms

**Figure 1:**
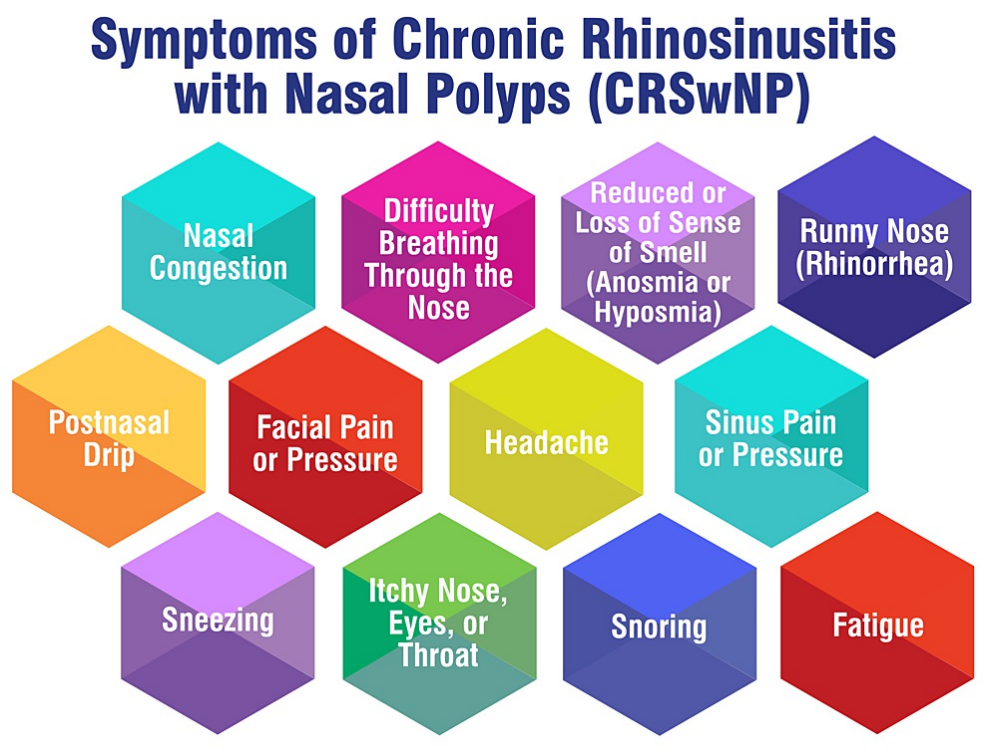
Symptoms of chronic rhinosinusitis with nasal polyps (CRSwNP) The image is created by the corresponding author

AR, characterized by symptoms such as nasal obstruction, rhinorrhea, nasal itching, and sneezing, is a prevalent condition marked by chronic inflammation of the upper airways mediated by immunoglobulin E (IgE). Patients afflicted with AR commonly endure nasal congestion, fits of sneezing, and itching sensations in the nose and eyes, coupled with watery rhinorrhea and a diminished sense of smell. Notably, dark circles under the eyes resulting from nasal congestion can indicate this condition [15]. NPs present distinct symptoms, including nasal obstruction, diminished sense of smell, nasal discharge, and facial pain or pressure. These benign growths within the nasal passages can induce blockages, congestion, and anterior or posterior nasal drip. Patients with NP may also experience facial discomfort or pressure, indicative of these growths within the nasal cavity [16]. Rhinosinusitis, encompassing inflammation of the sinuses and nasal passages, manifests with symptoms such as sinus pain, nasal congestion, and loss of smell. Facial discomfort or pressure is also prevalent among individuals affected by rhinosinusitis, reflecting the inflammation and congestion affecting the sinus cavities. These symptoms may vary in intensity and duration, often overlapping with those of AR and NP in individuals experiencing these conditions [17]. Figure 1 shows the symptoms of chronic rhinosinusitis with nasal polyps (CRSwNP).

Physical Examination

The physical examination for AR entails a comprehensive assessment of various areas to facilitate accurate diagnosis. This examination typically evaluates outward signs such as persistent mouth breathing, nasal mucosa swelling, and dark circles under the eyes resulting from nasal congestion. Moreover, the examination thoroughly assesses the nose, ears, sinuses, posterior oropharynx, chest, and skin to identify signs of AR [18]. Specific signs that may suggest AR include behaviors such as rubbing at the nose, the presence of a nasal crease, sniffling, throat clearing, and the presence of allergic shiners under the eyes [18]. During the nasal examination, clinicians may observe swelling of the nasal mucosa and thin secretions, while internal endoscopic examination aids in identifying structural abnormalities such as NP and ulcerations [18]. Additionally, although the ears typically exhibit normal findings in cases of AR, assessment for Eustachian tube dysfunction may be warranted. Examination of the sinuses for tenderness and evaluation of the posterior oropharynx for signs of postnasal drip are also vital components of the diagnostic evaluation [18].

Diagnostic Tools

Nasal endoscopy, or rhinoscopy, is a diagnostic tool to assess the nasal passages and sinuses, offering a direct visual examination of these structures through a magnified, high-quality view [19-21]. During a nasal endoscopy procedure, a healthcare provider inserts an endoscope-a thin, flexible, or rigid tube equipped with a camera at its end-into the nose to inspect the nasal cavity and sinuses for various issues such as polyps, mucus accumulation, masses, blockages, sinusitis, and nasal tumors [20-21]. This procedure enables visualization of the nasal and sinus structures, facilitating diagnosing conditions including nasal congestion, infections, polyps, tumors, nosebleeds, and loss of smell, among others [20-21]. Additionally, nasal endoscopy can be employed to obtain tissue samples for biopsy, remove polyps or masses, clear debris from the nasal passages, and evaluate the efficacy of treatments for nasal and sinus problems [20-21]. Overall, nasal endoscopy is a valuable diagnostic tool offering detailed insights into nasal and sinus anatomy, thereby aiding healthcare providers in identifying and addressing a broad spectrum of nasal and sinus conditions.

Imaging studies play a critical role in diagnosing NP. CT scans are commonly utilized to visualize the size and location of polyps deep within the sinuses, assisting in excluding other causes of nasal blockage [22]. These studies are pivotal in documenting the extent of sinus disease and providing essential information for precise diagnosis and treatment planning. Moreover, nasal endoscopy enables meticulous examination of the nasal cavity, including the clinically significant middle meatus in rhinosinusitis cases [23]. This technique offers a comprehensive perspective of internal nasal structures, facilitating the evaluation of allergic and inflamed mucosa, secretions, swelling in the middle meatus, and the presence and severity of NP [23]. Nasal endoscopy emerges as a valuable diagnostic tool furnishing detailed insights into nasal pathology, thereby contributing to effectively managing NP and associated conditions.

**Figure 2:**
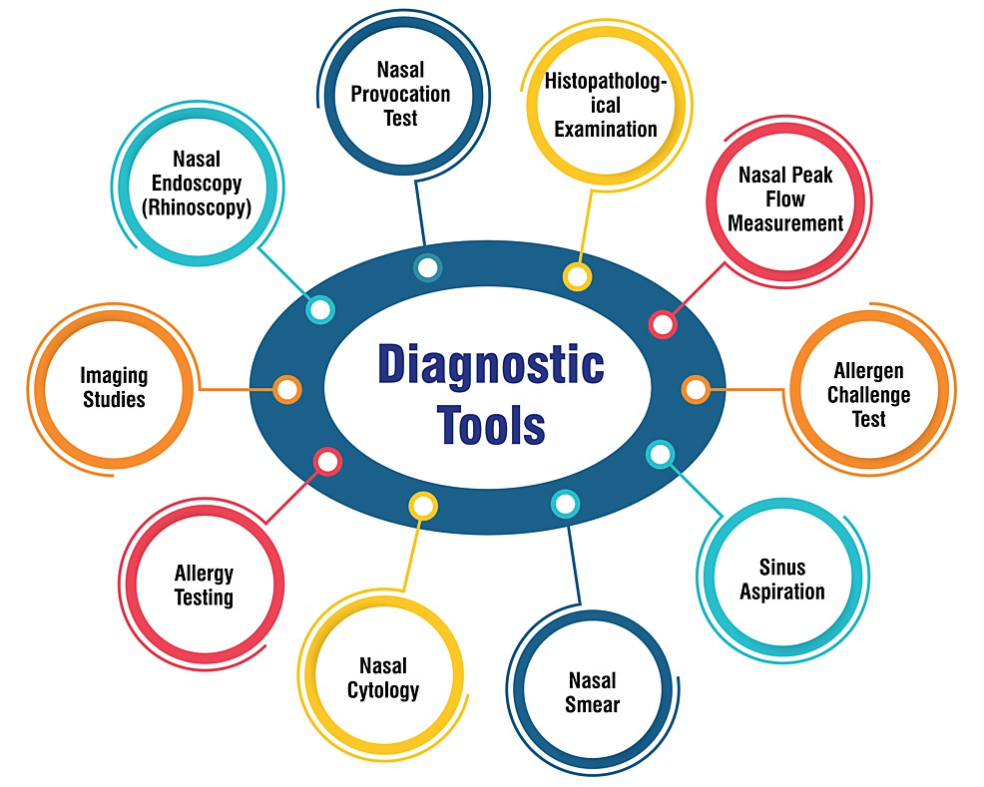
Diagnostic tools for allergic rhinitis with nasal polyps The image is created by the corresponding author.

Allergy testing, also known as sIgE blood tests, involves quantifying specific IgE antibodies in the blood, aiding in allergy diagnosis by detecting sensitization to allergens such as pollen, mold, food, and animal dander. These tests aid in confirming allergies, determining reaction causes, and identifying specific proteins that may trigger allergic reactions, enabling healthcare providers to devise tailored treatment plans based on precise allergen sensitivities [24]. Skin testing entails introducing a small amount of a suspected allergen into the skin using a skin allergy testing tool and observing the skin's response. A positive reaction is characterized by a wheal (swelling) and flare (redness around the wheal) within 20 minutes of application. This method helps identify allergic antibodies and pinpoint the exact allergens causing immune system reactions, facilitating personalized treatment plans and avoidance strategies tailored to specific triggers [24]. Intradermal testing involves injecting small amounts of allergens into the skin's superficial layers. This method aids doctors in identifying specific allergic sensitivities by observing the skin's reaction to allergens, typically manifesting as red, raised, itchy hives within minutes of exposure. Although temporary and self-resolving, these reactions furnish valuable information for effective allergy treatment and management [24]. Figure 2 shows the diagnostic tools.

Management of allergic rhinitis with nasal polyps

Pharmacological Therapies

Intranasal corticosteroids are a cornerstone in managing NP, offering standalone long-term therapy for mild cases and combination therapy with systemic corticosteroids and surgery for severe cases [25,26]. These corticosteroids play a pivotal role in alleviating rhinitis symptoms, enhancing nasal breathing, reducing polyp size, and mitigating their recurrence to some degree [25,26]. They effectively decrease eosinophil levels and mucin expression in NP, thereby relieving symptoms and improving nasal airflow resistance [27]. Supported by evidence from controlled trials, intranasal corticosteroids demonstrate efficacy in treating NP and CRSwNP [26,28]. First-line therapy recommendations stem from their ability to target the inflammatory process underlying NP, including the reduction of proinflammatory cytokines such as interleukin-5 and histamine, pivotal in NP pathogenesis [25,26].

Antihistamines significantly manage NP by alleviating symptoms like nasal sneezing, rhinorrhea, and nasal obstruction. Studies illustrate that antihistamines like cetirizine effectively diminish nasal sneezing and rhinorrhea in NP patients, with minimal impact on polyp size or number [29]. Moreover, antihistamines such as cetirizine exhibit few adverse effects when administered at double the recommended daily adult dose [29]. Commonly utilized antihistamines, including cetirizine, loratadine, and fexofenadine, aid in symptom management of AR and enhance the overall quality of life for NP patients [30]. These antihistamines block histamine action, a chemical released during allergic reactions, thereby mitigating symptoms like sneezing, itching, and runny nose associated with NP and AR [30].

Leukotriene receptor antagonists (LTAs) emerge as a potential strategy for managing CRSwNP, often linked with asthma and AR [31]. Agents such as montelukast, zafirlukast, and zileuton act by blocking cysteinyl-leukotriene receptors (CysLT1 and CysLT2), modulating eosinophil recruitment, bronchospasm, vasoconstriction, mucus secretion, and plasma exudation [32]. Meta-analyses of randomized controlled trials reveal LTAs' effectiveness in reducing nasal symptoms and polyp size in CRSwNP patients [31]. However, the clinical significance remains uncertain, as overall symptom improvement is modest, and the impact on quality of life remains unexplored [31]. When used as adjunctive therapy, Montelukast demonstrates statistically significant symptom improvement in CRSwNP patients compared to placebo. However, evidence quality could be improved, necessitating further research to define LTAs' optimal role in CRSwNP management [33]. Studies indicate montelukast's effectiveness in reducing nasal polyp size and improving symptoms in CRSwNP patients with asthma [34]. Additionally, LTAs effectively prevent postoperative nasal polyp recurrence in aspirin-sensitive rhinosinusitis (ASA syndrome) patients [35].Top of Form

Decongestants serve as a medication type offering relief for nasal congestion attributed to NP. They diminish swelling in the nasal passages, enhancing airflow and facilitating easier nasal breathing [36]. Available in nasal spray or oral form, decongestants can be obtained over the counter or via prescription. It's crucial to recognize that prolonged use of decongestants may result in rebound congestion, wherein nasal passages become more swollen after the medication's effects wear off, potentially fostering dependence on the medication to alleviate congestion. Consequently, decongestants should be utilized briefly and under healthcare provider supervision [36]. Other treatments for NP encompass nasal steroid sprays, which reduce inflammation and shrink polyp size, as well as surgical removal of polyps. These modalities can be combined with decongestants to alleviate nasal congestion and other associated symptoms [22].

Immunotherapy emerges as a practical approach for managing CRSwNP. Dupilumab, a monoclonal antibody targeting interleukin-4 and interleukin-13 signaling, demonstrates efficacy in enhancing subjective patient-reported outcomes and objective physician-evaluated metrics for CRSwNP [37]. NUCALA (mepolizumab) is a prescription medication used as an add-on maintenance treatment for CRSwNP in adults whose condition remains uncontrolled with nasal corticosteroids. NUCALA has shown efficacy in reducing nasal polyp size and congestion and diminishing the need for repeat surgeries when combined with existing nasal polyp treatments [38]. While evidence regarding the durability of biologics post-cessation of use remains limited, CRSwNP pathophysiologic mechanisms primarily involve a type 2 inflammatory reaction and aberrant eosinophilic infiltration. Recent research endeavors aim to refine the classification of this heterogeneous phenotype to enable the development of more targeted treatments [39]. Current management strategies involve medical and surgical interventions targeting generalized inflammation, including steroid nasal sprays, oral steroids, saline rinses, and antibiotics. Novel targeted biologics like dupilumab and mepolizumab offer promise in addressing different facets of the inflammatory pathway, although consensus on their optimal timing and utilization is yet to be established [39].

Surgical Interventions

Functional endoscopic sinus surgery (FESS) is a minimally invasive surgical intervention to address severe sinus conditions like chronic sinus inflammation, infections, and NP [40,41]. Utilizing nasal endoscopes- thin tubes equipped with lights; this procedure aims to unblock sinus openings and restore normal sinus function [40,41]. Typically conducted on an outpatient basis under general anesthesia, FESS yields significant symptom improvement for most patients post-surgery [40,41]. The procedure entails inserting a nasal endoscope into one nostril to visualize the sinuses and remove any obstructions, such as mucous membrane swelling, NP, or scar tissue [40,41]. Additionally, surgeons may rectify a deviated septum or diminish turbinate size to enhance nasal airflow [40,41]. Nasal post-surgery packing is often unnecessary, simplifying recovery [40,41]. Highly effective for treating chronic sinus conditions, FESS boasts significant symptom improvement rates ranging between 80% and 90% of patients post-surgery [40,41]. Nonetheless, like any surgical procedure, potential complications and risks exist, including bleeding, infection, or damage to surrounding structures [40,41]. Hence, patients should thoroughly discuss these risks with their healthcare provider before undergoing FESS.

Polypectomy emerges as a surgical intervention utilized to eliminate NP, particularly in the management of AR accompanied by NP [42,43]. While medical therapies for AR encompass antihistamines and topical nasal steroid sprays, NP management primarily involves medication and surgery [42,43]. NP medication comprises antibiotics, nasal steroid sprays, oral steroids, and saline rinses, with emerging therapies such as dupilumab and mepolizumab exhibiting promising outcomes in clinical trials [39,42]. Polypectomy involves surgical scissors or snares to excise polyps within the nasal passage [42]. Typically recommended for severe cases or when medication fails to yield desired outcomes, polypectomy may, however, be associated with polyp recurrence over time, the likelihood of which varies among individuals [42]. Management of CRSwNP adopts a multifaceted approach encompassing medical and surgical interventions [42,43]. Medical therapies target generalized inflammation and encompass steroid nasal sprays, oral steroids, saline rinses, and antibiotics [42]. Novel therapies like dupilumab and mepolizumab exhibit promise in targeting distinct facets of the inflammatory pathway, though optimal timing and usage guidelines remain undetermined [42].

Lifestyle Modifications

Allergen avoidance: Managing allergies, including AR, necessitates implementing allergen avoidance strategies. This involves taking measures to minimize exposure to allergens in our environment. This initial step is crucial for alleviating allergy symptoms. It entails avoiding foods or medications containing substances triggering allergic reactions, which demands meticulous attention to identifying hidden allergens [44]. Although allergen avoidance measures aim to reduce sensitizing allergen exposure and alleviate AR symptoms, their efficacy has been questioned, lacking a definitive demonstration of effectiveness [45]. While most preventive measures effectively decrease allergen exposure, and some surpass placebo in reducing AR episodes and improving patients' quality of life, their overall efficacy remains uncertain [45]. Environmental control measures may be inefficient against airborne pollens, necessitating identifying allergy triggers. Tracking the onset and severity of allergy symptoms can aid in identifying potential allergens, with consultation with healthcare providers recommended for individuals uncertain about their allergy triggers [46].

Nasal irrigation: Nasal irrigation, an age-old Ayurvedic practice, involves rinsing nasal passages with a saline solution to expel mucus and clear debris [47]. Employed alone or in conjunction with other therapies, nasal irrigation finds utility in various conditions, including chronic rhinosinusitis and allergies, particularly in children [47]. Effective methods ensure large-volume irrigation with positive pressure, facilitating comprehensive solution distribution in nasal and sinus cavities [47]. Nasal irrigation relieves symptoms associated with sinus infections, allergies, colds, flu, and even COVID-19 by removing dust, pollen, and debris and loosening thick mucus [48]. Proper device usage entails washing hands, ensuring device cleanliness and dryness, preparing saline rinse, adhering to manufacturer instructions, and device maintenance [48]. Distilled, sterile, or previously boiled water is recommended to mitigate the risk of potentially serious infections associated with tap water usage [48].

Humidification: Humidification is pivotal in managing airway conditions like AR and NP, achievable through active or passive means [49]. Active humidification involves heated humidifiers, while passive methods employ heat and moisture exchangers (HME) to retain heat and moisture [49]. Maintaining optimal moisture levels in nasal passages can alleviate nasal polyp symptoms, with humidifiers or gel bead humidification systems ensuring steady moisture release [50]. Industrial air humidification is utilized in production processes to maintain optimal air humidity levels, enhancing quality, product weights, and machine efficiency [51].

Emerging therapies and future directions

Biologic Agents

Biologic agents offer a contemporary therapeutic avenue for CRSwNP, with the FDA's approval of mepolizumab marking a significant milestone as the first biologic agent sanctioned for this condition [52]. Alongside mepolizumab, dupilumab and omalizumab are the two other FDA-approved biologics targeting type 2 inflammation in NP [53]. Indicated for patients with bilateral NP, these biologics have demonstrated notable improvements in symptoms and quality of life [52]. The utilization of biologics in CRSwNP represents a swiftly progressing field, with ongoing research aimed at pinpointing the most suitable candidates for these therapies and refining the timing of treatment initiation. A proposed study design outlines gathering data regarding the timing of biologic therapy initiation, focusing on patients with CRSwNP who have undergone previous surgical intervention [52]. The study aims to compare the effects of initiating biologic therapy at various time points post-surgery. In clinical settings, identifying eligible patients for biologics in CRSwNP remains pivotal [52].

Immunomodulatory Therapies

Immunomodulatory therapies have demonstrated considerable promise in managing CRSwNP by targeting specific inflammatory pathways implicated in its development and progression. Among these therapies are biologics, monoclonal antibodies designed to target particular cytokines or receptors integral to the inflammatory cascade. Notable biologics in this realm include omalizumab, dupilumab, and mepolizumab, each showcasing clinical efficacy in treating CRSwNP [54,55]. Omalizumab, an anti-IgE antibody, can diminish nasal polyp size and ameliorate symptoms in CRSwNP patients [54,55]. Dupilumab, an anti-IL-4RA antibody, has been found to reduce nasal polyp size, enhance symptoms, and lessen the necessity for surgery in CRSwNP patients [54,55]. Similarly, mepolizumab, an anti-IL-5 antibody, has effectively reduced nasal polyp size and improved symptoms in CRSwNP patients [54,55]. Other promising immunomodulatory therapies for CRSwNP include anti-IL-5 therapeutics like reslizumab and mepolizumab [54], which target IL-5, a pivotal player in eosinophil maturation and activation, crucial components of CRSwNP's inflammatory response.

Precision Medicine Approaches

Precision medicine represents a groundbreaking approach to disease treatment and prevention, acknowledging each person's variances in genes, environment, and lifestyle [56,57]. This emerging paradigm aims to revolutionize diagnosis, treatment, and prevention by tailoring approaches to be more personalized, proactive, predictive, and precise [56]. Unlike traditional one-size-fits-all methods, precision medicine recognizes the distinct characteristics of each individual, encompassing their genetic composition, environmental influences, and lifestyle choices [56,57]. The overarching objective is administering appropriate treatments to the right patients at the optimal time, thereby enhancing survival rates and minimizing exposure to adverse effects [56]. The strides made in precision medicine have yielded significant breakthroughs, leading to the development of FDA-approved treatments customized to an individual's genetic makeup or the genetic profile of their tumor [56]. Leveraging big data, mobile health, imaging, artificial intelligence, social engagement, and networking, precision medicine endeavors to compile comprehensive datasets capable of formulating preventive treatment plans for individuals and communities [56]. These datasets can aid in predicting wellness risks, disease progression, and responses or resistance to therapy [56]. Precision medicine is the nexus between individuals, their environments, health and illness markers fluctuations, and social and behavioral determinants over time [56]. It scrutinizes the multifaceted factors composing individuals and populations and examines how these elements evolve and interact over time [56]. Within precision medicine, diseases can be categorized into various subtypes through molecular subtyping utilizing diverse biomarkers and omics data, clinical subtyping based on electronic health records (EHRs), and consideration of environmental, social, and behavioral influences [56]. This approach facilitates the development of tailored therapies for numerous subtypes and sub-subtypes of prevalent diseases [56].

Gene Therapy

Gene therapy is an emerging frontier in managing CRSwNP. While biologics like monoclonal antibodies have shown promise in alleviating symptoms such as nasal blockage and impaired sense of smell, as well as reducing polyp size, there are limitations to conventional treatment approaches, notably the recurrence of NP [58,59]. Recent research has unveiled several potential therapeutic targets for CRSwNP, including variations in the thymic stromal lymphopoietin gene that exhibit an association with CRSwNP dependent on gender and the presence of NP [60]. Furthermore, single-cell RNA sequencing has uncovered alterations in epithelial cells, fibroblasts, and critical genes implicated in CRSwNP [61]. Gene therapy entails leveraging genes to either treat or prevent disease. In the context of CRSwNP, gene therapy could involve deploying genes to modulate the immune response, mitigate inflammation, or facilitate tissue repair. For instance, gene therapy might entail delivering genes encoding anti-inflammatory cytokines or enzymes responsible for degrading the extracellular matrix of NP.

Role of Microbiome in Management

**Figure 3:**
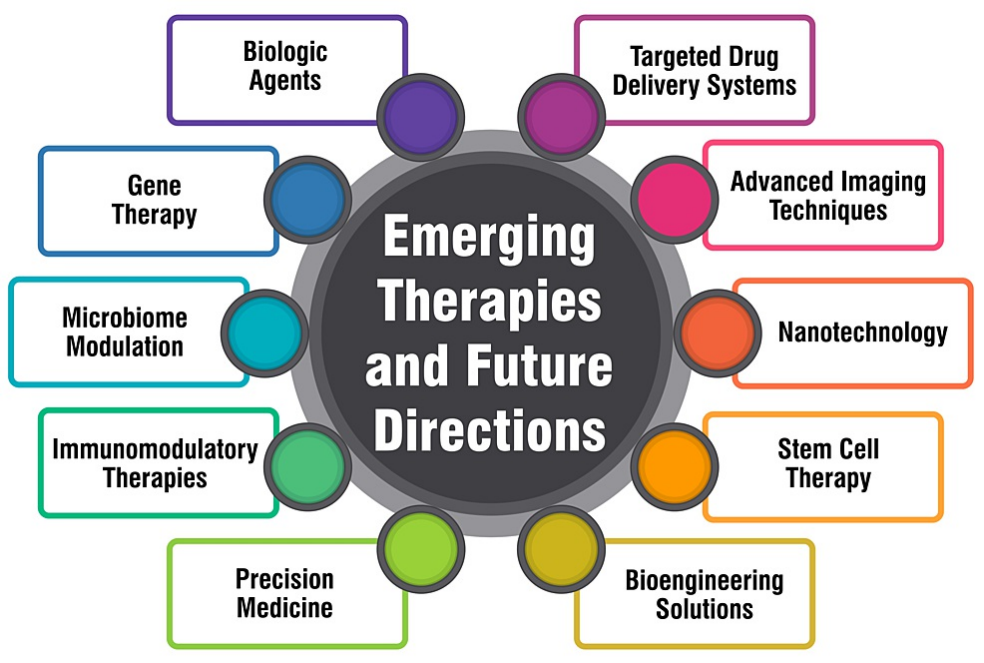
Emerging therapies and future directions for treating allergic rhinitis with nasal polyps The image is created by the corresponding author.

The microbiome plays a pivotal role in managing chronic rhinosinusitis (CRS) with NP, referring to the collective community of microorganisms, including bacteria, viruses, and fungi, that inhabit the sinuses. Recent research has highlighted significant differences in the sinus microbiome between CRS patients and healthy individuals [62-64]. In CRS patients, the sinus microbiome typically exhibits reduced microbial diversity alongside an elevated presence of specific bacterial species, notably Staphylococcus aureus and Haemophilus influenzae [62-64]. These alterations in the microbiome composition can contribute to the inflammatory processes and immune responses underlying CRS, ultimately contributing to the development of NP [62-64]. Consequently, comprehending the microbiome's role in CRS with NP is pivotal for devising novel therapeutic approaches. One potential strategy involves introducing probiotics, live bacteria, or yeast into the body to confer health benefits [62-64]. Probiotics have demonstrated the ability to modulate the immune response and diminish inflammation in the sinuses, potentially ameliorating CRS severity and mitigating nasal polyp formation [62-64]. Another avenue of exploration is fecal microbiota transplant (FMT), which entails transferring stool from a healthy donor to a patient to restore microbiome equilibrium [62-64]. While FMT has shown effectiveness in treating certain gastrointestinal disorders, its potential application in CRS with NP garners increasing interest [62-64]. Figure 3 shows emerging therapies and future directions.

## Conclusions

This comprehensive review has highlighted the intricate relationship between AR and NP, offering insights into their shared mechanisms, clinical presentations, and therapeutic approaches. Notably, allergic inflammation is a significant contributor to the development and exacerbation of NP, underscoring the importance of identifying and managing AR in affected individuals. The findings underscore the need for a multidisciplinary approach to patient care, involving collaboration among allergists, otolaryngologists, and respiratory specialists to tailor treatment plans to the individual patient's needs. Moreover, the review emphasizes the potential of emerging therapies, such as biologic agents and immunomodulatory treatments, in improving treatment outcomes and addressing unmet needs in patient care. Future research endeavors should prioritize elucidating the underlying mechanisms driving these conditions and evaluating novel therapeutic strategies, including precision medicine approaches and microbiome-targeted interventions, to advance our understanding and management of allergic respiratory diseases. By leveraging these insights and implementing evidence-based practices, we can strive towards enhancing outcomes and quality of life for individuals suffering from AR and NP.
